# Children's Hearing Health Panorama in the Unified Health System in the state of Sergipe

**DOI:** 10.1590/2317-1782/20232021197en

**Published:** 2023-12-18

**Authors:** Josilene Luciene Duarte, Kelly da Silva, Fabiana Cristina Carlino, Maria Victória dos Anjos Souza, Greicielly da Silva Pereira Vieira, Ana Maria Carregosa, Sulamita Cysneiros das Chagas Santos

**Affiliations:** 1 Departamento de Fonoaudiologia, Universidade Federal de Sergipe - UFS - Lagarto (SE), Brasil.; 2 Clínica Otocenter - Aracaju (SE), Brasil.

**Keywords:** Neonatal Screening, Hearing Loss, Early Diagnosis, Rehabilitation, Quality Indicators, Health Care

## Abstract

**Purpose:**

To describe the panorama of children’s hearing health in the Unified Health System of the state of Sergipe.

**Methods:**

A quantitative and retrospective study consisting of four steps: 1) Search the National Registry of Health Establishments of institutions affiliated to the Health Unic System in the state of Sergipe that perform obstetric services and hearing health services; 2) Collecting Neonatal Hearing Screening (NHS) coverage data through DATASUS (from 2012 to 2020); 3) Data collection from medical records of institutions with obstetrics and that perform NHS; and 4) Interview with the guardians of children undergoing auditory rehabilitation. The results were summarized using descriptive statistics (absolute and relative frequency, measures of central tendency, and dispersion).

**Results:**

Only one out of the 29 establishments with obstetrics performs NHS. Two of the Hearing Health Reference Centers (HHRC) are qualified for cochlear implants and two Specialized Centers are qualified for Rehabilitation. From 2012 to 2020, NHS coverage in the state was less than 40%, and when performed in the maternity ward, there were no referrals for Brainstem Auditory Evoked Response (BERA) and audiological diagnosis. The HHRC showed considerable coverage and a lower evasion rate to perform BERA, with a diagnosis rate of 4.8%. The mean time from the NHS to rehabilitation was longer than recommended.

**Conclusion:**

NHS coverage must be increased, adjusting the hearing health network to articulate the different levels of care, and reducing the time for identification, diagnosis, and start of rehabilitation.

## INTRODUCTION

More recently, public policies for hearing health have been advancing to favor the early diagnosis and rehabilitation of hearing loss. The Multi-professional Committee on Hearing Health (COMUSA - *Comitê Multiprofissional em Saúde Auditiva*)^(1)^, based on the international standards of the Joint Committee on Infant Hearing (JCIH)^(2)^ of 2007, created the quality indicators for Neonatal Hearing Screening (NHS) in 2010. The aim is to standardize the Risk Indicators for Hearing Loss (RIHL) and achieve the objectives of hearing health programs, which include the full development of oral language.

These indicators were the basis for the Ministry of Health to create the Guidelines for Attention to Neonatal Hearing Screening^(3)^ in 2012. In 2020, after updating the JCIH^(4)^ document (2019), COMUSA^(5)^ published a technical note adding new RIHL and highlighting the existing recommendations for performing Evoked Otoacoustic Emissions (EOAE) procedures for children without RIHL and the Auditory Evoked Potential of Brainstem - Automatic (BAEP-A) for children with risk indicators.

This update added the following minimum goals for NHS coverage: performance in 95% of neonates before hospital discharge or at most within the first month of life; NHS retest, when necessary, 15 days after hospital discharge; less than 4% of referrals for diagnosis; completion of diagnosis by the third month of life in 90% of cases; the start of sound amplification in 95% of cases of permanent bilateral hearing loss, within one month after diagnosis confirmation, and the start of auditory rehabilitation between the third and sixth month of the child’s life^(1-6)^.

National studies covering different regions of the country have shown the need to increase NHS coverage and meet the established goals, in addition to pointing to a regional disparity, with better results for the Southeast and South of the country and worse in the North and Northeast^(7-12)^.

Additionally, from 2008 to 2011, national studies found a 208% growth in NHS coverage. The South and Northeast regions had the greatest growth rates^(13)^, with the South presenting an even higher rate than estimated (189%), in addition to the highest proportional increase (65%). The North region, in turn, despite having half the coverage (54.5%), emerged as the second highest increase (58%)^(14)^, which indicates that before such an increase its coverage was far below the rest of the country. Until 2015, the North and Northeast regions had a higher percentage of places without any NHS coverage^(6)^.

In this sense, there is a clear need for studies that demonstrate the organization of the hearing health network for the pediatric population in different regions of Brazil. Research must show whether the regulations are being respected and discuss improvement possibilities and the overcoming of obstacles. Therefore, this study aims to describe the panorama of children’s hearing health in the Unified Health System (in Portuguese SUS) in the state of Sergipe.

## METHODS

This is an analytical study based on a quantitative, retrospective, cross-sectional approach and approved by the Research Ethics Committee (Opinion number 4,293,898; CAAE 92530218.7.0000.5546). This research covered the following four steps: 1) Survey of institutions affiliated to the SUS in the state of Sergipe that perform obstetric services and hearing health institutions, at all levels of complexity, in the National Registry of Health Establishments (CNES); 2) Collecting NHS coverage data through DATASUS from 2012 to 2020; 3) Data collection from medical records of institutions with obstetrics and/or that perform NHS; and 4) Interview with parents and/or guardians of children undergoing auditory rehabilitation.

### Survey of institutions affiliated with the SUS in the CNES

Initially, we performed an active search in the National Register of Health Establishments (CNESNet) - through the address of the CNES^(15)^ -, for information on health establishments affiliated with the SUS that provide obstetric care (in addition to offering the Universal Neonatal Hearing Screening - UNHS) and that promoted actions aimed at hearing health from primary to specialized care.

### Collecting NHS coverage data through DATASUS

We searched the DATASUS for information from 2012 (the year of NHS start in the state of Sergipe) to 2020. The number of Live Born Neonates (LBN) was provided by the Information System on Live Births - SINASC, whereas the number of NHS was provided by the Outpatient Information System (SIA-SUS - *Sistema de Informações Ambulatoriais*). Table 1 shows the procedure codes used for the surveys in the SIA-SUS.

**Table 1:** Procedure codes used in the search in the Outpatient Information System of the Unified Health System (SIA-SUS)

Researched procedures	Code of procedures
Evoked otoacoustic emissions for hearing screening (little ear test)	0211070149
Auditory Evoked Potential for hearing screening (little ear test)	0211070270
Assessment for the differential diagnosis of hearing loss*	0211070106
External behind-the-ear hearing aid type C^1^^*^	0701030143
Unilateral Cochlear Implant Surgery*	0404010571
Bilateral Cochlear Implant Surgery	0404010580

* Indicates procedures also performed in specific groups, with individuals older than three years. ^1^Indicated for the children's age group due to the versatility provided by the algorithms and electroacoustic characteristics.

NHS coverage in the state was calculated based on data collected at DATASUS considering the number of Live Born Neonates (LBN) subtracted from the number of newborns who underwent NHS (using Otoacoustic Emissions or Brainstem Auditory Evoked Potential (BAEP) (LBN - (NHS EOA + NHS BAEP). The percentage of NHS coverage was calculated by the formula ([NHS EOA + NHS BAEP] /LBN) * 100.

### Gathering data from medical records

Upon gathering the information on the health establishments by the CNESnet, the State Department of Health indicated four institutions accredited by the SUS that effectively carry out hearing health care in the state of Sergipe: the Association of Maternal-Infant Assistance and Protection of Sergipe (Maternidade Zacarias Junior), the Rehabilitation Center Specialized of Sergipe (CER III Dona Maroca), the Hearing Health Reference Center - CRSA *Centro de Referência em Saúde Auditiva* (Hospital São José), and the University Hospital of the Federal University of Sergipe on Campus of São Cristóvão (HU-UFS).

An invitation letter was sent to the managers of these four institutions, in addition to the ICF and a Google Form for collecting information regarding the number of NHS, auditory diagnosis, granting of electronic hearing devices, and speech-language therapy. The following three services agreed to participate in the research: the Lagarto Maternal and Child Assistance and Protection Association (Maternidade Zacarias Junior), CER III, and CRSA (Hospital São José). However, CER III was disregarded due to its recent authorization and absence of a service demand flow in hearing loss. Thus, 3,741 records were analyzed at the maternity hospital and 11,400 at the CRSA.

The percentage of NHS evasion at the maternity hospital was calculated considering the formula (newborns who did not undergo the NHS / total number of medical records analyzed) * 100. For the CRSA, the percentage was calculated by (newborns who did not return for NHS retest / total number of newborns who underwent NHS) * 100.

### Collecting primary data via telephone interview

Data on the average time between the NHS process and rehabilitation were gathered from the information of 25 individuals who were undergoing speech-language therapy at CRSA. Via telephone interview, the parents were instructed on the objectives of the study and on free and spontaneous will upon the interviewer reading the letter of information and the ICF to the research subject.

Parents were asked to provide the following information: the child’s age at NHS performance; the time between the NHS test and retest; the child’s age at the first consultation to start the audiological diagnosis (at the reference center); the time between the start and the close of the diagnosis; the child’s age at the first appointment for selecting the hearing electronic device; the time between the diagnosis and device selection, the child’s age at device adaptation, the time between selecting and adapting the device; and the child’s age at the start of speech-language therapy.

### Data tabulation and analysis

Data were entered into Microsoft Office Excel spreadsheets. To summarize the results, descriptive statistics were performed by calculating the following: relative and absolute frequencies for categorical results, measures of central tendency (mean) and dispersion (standard deviation) for quantitative data, and estimation of NHS coverage and evasion indicators.

## RESULTS

### Survey in the CNES of institutions affiliated with the SUS

Data collected from the CNESNet for the state of Sergipe indicated 29 establishments that offer obstetric services through the SUS. Only two (6.9%) out of these perform NHS (one in the city of Lagarto and the other in Aracaju). Newborns born in two public maternity hospitals in the capital (Nossa Senhora de Lourdes and Santa Izabel) were referred to NHS in the only two CRSA qualified to perform cochlear implants (one at the São José Hospital and the other at the University Hospital of the Federal University of Sergipe). There are still two CERs in the state, CER IV, in the Aracaju region, and CER III, in the Lagarto region, that cover every service from screening to hearing rehabilitation and provide an Individual Sound Amplification Device (ISAD).

### Gathering NHS coverage data through DATASUS

Table 2 shows the data collected from the DATASUS for the following elements: the number of the LBN; the neonatal auditory screenings; the auditory evaluation for the studied age group; and the supply of hearing electronic devices for the studied age group for the state of Sergipe from 2012 to 2020. It is worth highlighting that age groups cannot be distinguished for the hearing assessment and the supply of electronic hearing devices since the codes for the group under three years apply to procedures that can be used for an older age group. In this case, the percentage was presented only for the NHS.

**Table 2:** Overview of child hearing health care in the state of Sergipe based on DATASUS

Year	LBN	EOAE NHS (without risk) (n, %)	BAEP NHS (with risk) (n, %)	Total NHS (%carried out)	Assessment for Diagnosis^*^ (n)	ISAD type C^**^ (n)	CI^***^ (n)		
2012	27.354	3.801 (13.9%)	200 (0.7%)	4.001 (14.6%)	25	174	----	----	----
2013	27.183	5.999 (22.1%)	67 (0.2%)	6.066 (22.3%)	26	258	----	----	----
2014	27.502	8.523 (31%)	224 (0.8%)	8.747 (31.8%)	48	169	13	----	13
2015	27.797	8.693 (31.3%)	183 (0.7%)	8.876 (31.9%)	172	88	18	----	18
2016	25.702	8.240 (32.1%)	118 (0.5%)	8.358 (32.5%)	56	88	17	----	17
2017	33.867	8.764 (25.9%)	121 (0.4%)	8.885 (26.2%)	131	101	16	7	23
2018	34.256	11.536 (33.7%)	115 (0.3%)	11.651 (34.0%)	27	54	7	6	13
2019	32.697	8.599 (26,3%)	146 (0.4%)	8.745 (26.7%)	51	88	7	7	14
2020	31.784	6,393 (20.1%)	208 (0.7%)	6.601 (20.8%)	18	87	6	2	4
2012-2020	240.788	70.548 (29.3%)	1.382 (0.6%)	71.930 (29.9%)	554	1107	68#	22##	102###

* SUS code 0211070106 (assessment for differential diagnosis of hearing loss), which includes assessment for children under 3 years old;

** Type C hearing aids refers to the type of technology which is indicated for children;

*** CI with pediatric and adult indication;

# SUS code 0404010148 (cochlear implant);

## SUS code 0404010571 (unilateral cochlear implant surgery);

### SUS code 0404010580 (bilateral cochlear implant surgery)

**Caption:** LBN = live-born neonates; EOAE = evoked otoacoustic emissions; NHS = neonatal hearing screening; BAEP = brainstem auditory evoked potential; n = number of cases; % = percentage of cases; ISAD = individual sound amplification device; CI = cochlear implant

Based on DATASUS data from 2012 to 2020, the NHS coverage in LBN was 27.7%.

### Gathering data from medical records

**Figure 1:**
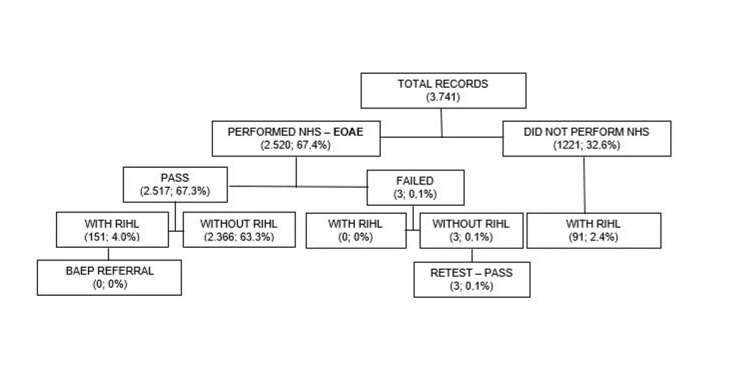
Absolute values and percentages of medical records analyzed in the maternity of the Lagarto (SE) region, according to the flow of events of the NHS Program performed only EOAE-T, from January/2013 to October/2020

Data were collected from 2013 to 2020 in the maternity hospital, with a monthly average of 210 LBN, and from 2018 to 2020 in the CRSA. Figure 1 shows a flowchart of the medical records analyzed at the maternity hospital under study, according to the NHS outcomes.

**Caption:** NHS = Neonatal Hearing Screening; EOAE = Evoked Otoacoustic Emissions; RIHL = Risk Indicator for Hearing Loss; BAEP = Brainstem Auditory Evoked Potential

The data collected show a NHS coverage of 67.4% in the maternity ward from 2013 to 2020. A data analysis covering the last three months showed that 36 (5.8%) out of the total of 625 LBN, did not undergo NHS, thus showing a coverage of 94.2%. All neonates underwent NHS with EOAE-T and none of them were referred to perform BAEP in those with RIHL (6.4% of the NBs during the study period).

**Figure 2:**
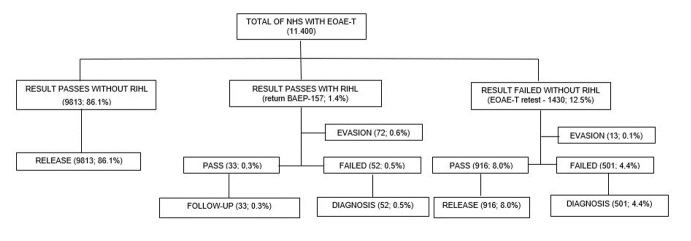
Absolute values and percentages of newborns screened with EOAE-T and BAEP at the Reference Center for Hearing Health (CRSA) in the Aracaju (SE) region from April/2018 to March/2020

Figure 2 shows a flowchart of the records analyzed in the CRSA according to the NHS outcomes.

**Caption:** NHS = Neonatal Hearing Screening; EOAE = Evoked Otoacoustic Emissions; RIHL = Risk Indicator for Hearing Loss; BAEP = Brainstem Auditory Evoked Potential

As in the maternity ward, NHS is performed in the CRSA using otoacoustic emissions evoked by transient EOAE-T stimuli. For the BAEP in newborns with RIHL, a return appointment is scheduled regardless of the NHS result. Children who fail the NHS but do not have RIHL return for the retest with EOAE-T only. The evasion rate in returning to perform the BAEP in newborns with RIHL was 45.8% and the evasion rate of those who failed the NHS and did not have RIHL was 0.9%.

The total avoidance rate for NHS in the maternity ward after hospital discharge from 2012 to 2020 was 32.6%. Upon performing the NHS at CRSA, the total dropout rate for NHS return was 0.6%.

### Gathering primary data through interviews

A telephone interview was conducted with parents/guardians of 25 patients who were undergoing speech therapy for auditory rehabilitation at CRSA in 2020. This type of analysis allows to respect the initial period of 2012 as many children who were undergoing therapy in this service were born before. Table 3 shows the results of 25 patients.

**Table 3:** Patients’ age when using devices (cochlear Implant and/or Individual Sound Amplification Device (ISAD) at the time of NHS, diagnosis, adaptation of electronic device, and start of speech-language therapy at the Reference Center for hearing health care

		AGE at the time of				DEVICE
ID	DB	NHS	Diagnosis	Adaptation	Therapy	
1	04/11/04	NP	168	180	180	CI
2	06/05/06	NP	30	84	72	ISAD and CI
3	27/07/07	22	22	23	23	ISAD and CI
4	13/10/07	4	10	24	36	ISAD and CI
5	14/11/08	16	16	18	16	ISAD and CI
6	02/02/09	16	18	20	112	CI
7	07/07/11	Birth	19	32	24	ISAD and CI
8	22/10/11	NP	24	36	36	CI
9	07/11/11	Birth	15	15	17	ISAD and CI
10	28/12/11	NP	17	20	20	CI
11	16/01/12	NP	48	48	48	ISAD and CI
12	30/09/12	NP	20	36	36	ISAD and CI
13	26/11/13	Birth	19	48	36	ISAD and CI
14	08/01/14	Birth	20	36	36	ISAD
15	01/03/15	Birth	1	2	4	CI
16	18/04/15	18	24	27	38	ISAD and CI
17	14/12/15	12	12	18	18	CI
18	21/01/16	Birth	12	18	37	CI
19	09/04/16	12	16	36	24	CI
20	26/08/16	NP	36	36	3	ISAD
21	22/10/16	22	22	29	24	ISAD
22	22/02/17	4	24	24	24	CI
23	14/04/18	2	12	19	19	ISAD
24	18/04/18	8	8	29	29	ISAD
25	24/05/19	4	24	28	28	ISAD

Caption: ID = identification; DB = date of birth; NP = not performed; NHS = neonatal hearing screening; CI = Cochlear Implant; ISAD = Individual Sound Amplification Device

Fifteen out of the 25 individuals were born after 2012, when the NHS started in the Sergipe state, and three out of these did not undergo the NHS and were referred to the CRSA from primary care for audiological evaluation based on a family complaint delay regarding the development of auditory and language functions. Considering each stage of the hearing health program for this group of individuals from 2012 to 2020, the average time (± standard deviation) in months per stage was 6.8 (± 7.6) for NHS; 16.1 (±7.3) for diagnosis; 26.1 (±11.5) for supplying electronic hearing aids and 26.4 (±9.9) for starting speech-language therapy.

## DISCUSSION

The main results of this research indicate that the NHS coverage and dropout rates are above the recommended level in the Sergipe State, taking a long time between diagnosis and auditory rehabilitation. Therefore, such a scenario indicates challenges to be faced by public services.

Hearing health care in the state of Sergipe, from NHS to rehabilitation, started in 2012, with NHS initially carried out only by the CRSA. The state is divided into seven health regions, with NHS offered almost entirely by the Aracaju and Lagarto regions. Some regions, such as Estância, Propriá and Nossa Senhora do Socorro (NSS), also adhered to NHS, but DATASUS records show low coverage in 2020. These data demonstrate that there is inequality in the supply of this service in the state.

Only one maternity hospital out of the 29 establishments that offer obstetrics offers NHS (in the city of Lagarto) and the only two CRSA in the state, which are in the Aracaju region, only receive newborns from two maternity hospitals in the Aracaju region. Thus, newborns born in other maternity hospitals are not referred to the NHS. DATASUS data warn that although approximately 2,500 children are born per month in the state, only 700 NHS are performed per month, revealing a low NHS coverage in this region of Brazil^(6)^. Contrary to expectations^(13)^, no increase in this coverage was observed over the years in the state of Sergipe.

Still based on the results from the DATASUS, the NHS coverage is far below recommended and represents the biggest challenge for the studied state in complying with regulations^(1-5)^ of public policies on hearing health since the NHS is the gateway to the effectiveness of the program.

The general analysis of the results revealed that to achieve the objectives of the public policies for hearing health proposed by the Ministry of Health, more units and/or maternity hospitals must be accredited to carry out NHS in the state. The coverage found, varying from 14.0% to 34.0% between 2012 and 2020 is still much lower than the recommended, which is 95.0% of LBN (1, 5). In addition, it is worth highlighting the importance of these centers being articulated with the CRSA and the CERs so that newborns who need monitoring and audiological diagnosis can be referred and scheduled.

The equipment indicated for performing NHS is the Evoked Otoacoustic Emissions (EOAE) and the Brainstem Auditory Evoked Potential (BAEP)^(2-5)^ devices. Since 2012, the BAEP has been listed by the Ministry of Health as mandatory for newborns with RIHL (3). Many institutions that perform NHS still only have EOAE, either by transient stimuli (EOAE-T) or by distortion product (EOAE-PD) due to the high cost of the BAEP equipment, which hampers its acquisition by some public institutions^(12)^. A strategy for this problem would be to refer newborns with RIHL to institutions accredited by the SUS that perform BAEP and are part of the specialized network.

As to the NHS performance, the EOAE-T is used both in the maternity ward and the CRSA. In the CRSA, regardless of the NHS result (Pass or Fail) newborns with RIHL attend a return visit to the institution for the BAEP. Neonates without IRDA but with NHS failure are subjected to the retest with EOAE-T. These findings corroborate some literature studies in which NHS is performed using EOAE-T regardless of the newborn's condition^(12,16)^. However, it is worth pointing out that the EOAE-T is not sensitive to detect retro-cochlear hearing loss; therefore, this type of hearing alteration might not be detected early, which opposes to the NHS objectives^(1-5)^.

Such a scenario is reinforced by the lack of referral to the BAEP for newborns with RIHL who were born in the maternity ward. Furthermore, in the CRSA, most of the newborns with RIHL who were subjected to the NHS using the EOAE-T returned for the BAEP, thus reinforcing the hypothesis of newborns with retro-cochlear alteration in this group. This hypothesis could be confirmed by accessing the diagnostic evaluation, which was not possible given the large sample and the lack of a database digitized by the institution.

It is also worth highlighting the BAEP performance already in the first NHS test for newborns with RIHL since the BAEP evasion rate for neonates who passed the EOAE-T is almost half of the cases, significantly higher than that of those who failed in otoemissions.

Another finding that drew attention regarding the NHS in the maternity ward was the high rate of the “pass” result using the EOAE-T associated with the non-referral to the BAEP for diagnosis and audiological follow-up in the case of RIHL. The literature describes that even the programs that can perform NHS universally present a rate of referral for diagnosis around 0.3 to 1.8%^(7,11,16)^, and it is recommended not to exceed 4.0%^(1,3)^.

It is worth noting that programs that achieved a rate of adherence to NHS in the maternity hospital like that found in this study (around 65.0%) had a referral rate of around 2.0%^(8,9)^. Thus, despite complying with Law nº 12.303^(17)^, the Lagarto maternity hospital does not provide the necessary operationalization to be inserted in the context of hearing health care.

Differently, in the CRSA the “pass” result rate was approximately 86.0%, corroborating with other studies that report data from 75.6 to 88.3%^(18-20)^, as well as referral for follow-up due to RIHL 0.3% and diagnosis 4.8%

The dropout rate observed herein approaches those found in the literature, ranging from 9.0% to 40.0% in various studies^(7,10,11,21)^. The NHS performed in the CRSA indicated a dropout rate for the return of 0.7%, possibly because the parents/guardians understood the need to perform the test for the early detection of hearing loss, which is a factor to be considered for the program^(22)^. Thus, guidance and contact with the newborn’s family, regardless of where the NHS was performed, can help reduce the rates of evasion from NHS and the program as a whole.

Since none of the centers where consent was obtained to carry out the research had a database, the hearing health program could not be managed. Such a finding corroborates the literature reports regarding programs in different regions of the country^(12)^. According to the COMUSA^(1)^ and the Ministry of Health^(3)^, databases favor the measures of quality indicators and reveal the overview of the hearing health program in terms of retest percentages, referrals for monitoring the function of hearing loss, audiological diagnosis, supply of electronic hearing devices, and speech-language therapy.

The parents/guardians were interviewed via telephone based on a questionnaire regarding the time between the steps from the NHS to speech-language therapy. Such an instrument was applied due to the lack of a database that verified whether the hearing health programs met the international criteria to minimize the effects of sensory deprivation on the neuroplasticity of the auditory system. The initial proposal was to collect information since 2012 (the year NHS started in the state); however, only 25 individuals were enrolled in speech-language therapy at the collection institution, thus requiring assessment of all periods. According to the Ministry of Health^(3)^ and the COMUSA^(1,5)^, the NHS (test and retest) must be performed in the first month of life, the diagnosis by the third month and rehabilitation must start by the sixth month. Our data showed that the time between steps is much longer than recommended.

The assessment for diagnosing hearing loss requires several stages of procedures; therefore, it is often not possible to complete it in a single session, thus resulting in a delay in this stage of the process. The same might occur from the test stage to the selection and supply of electronic hearing devices. However, according to our data, it is clear that once the newborn reaches this level of care, there is no considerable delay for the diagnosis, with more time spent in the test/selection/adaptation of electronic devices and hearing aids.

Such a long period happens because the SUS often does not have the devices readily available. To mitigate the damage caused by sensory deprivation in this period of acquisition of auditory and language skills, this service includes the patient in therapy, even before supplying electronic hearing devices.

It is worth highlighting that a well-structured hearing health program is necessary for the success of public policies in hearing health, not to compromise the main objective of the NHS and the hearing health program: detection, diagnosis and early intervention. The goal is to build auditory and language functions within the period of greater neuronal plasticity for them to develop as in normal-hearing children.

It is worth reinforcing that investment in public policies in hearing health prioritizes orality, and sign language will only be an alternative for families that are not interested in the development of orality, either due to family and/or cultural factors or for those individuals with comorbidities that prevent oral language from developing.

As for the limitations of the study, the lack of a database for managing and operating the local hearing health program, associated with the very low amount of information in the medical records, hampered gathering the information required to measure the long-term quality indicators of services for the studied state. Long-term studies are needed to monitor newborns individually, from NHS to auditory rehabilitation, to detect the actual difficulties of programs in this region and promote actions and solutions that lead the state to evolve in public health policies.

## CONCLUSION

The overview of children’s hearing health in the state of Sergipe describes actions at different care levels that require adjustments for the early detection and treatment of hearing loss. The NHS coverage is significantly below that recommended by the competent bodies, requiring supply in the different municipalities of the state and greater technological investment, since most NHSs are performed only using the EOAE. In addition, the hearing healthcare network must be articulated for the patient to access different care levels and to reduce the time for identification, diagnosis, and rehabilitation start, which, as found herein, is longer than recommended.
