# Supplementary material for: Children's Hearing Health Panorama in the Unified Health System in the state of Sergipe
Source: Codas. 2023 Dec 18;36(1):e20210197. doi: 10.1590/2317-1782/20232021197en (PMC10750856; doi:10.1590/2317-1782/20232021197en)
Supplement: Translation

# Translation: Panorama da Saúde Auditiva infantil no Sistema Único de Saúde no estado de Sergipe

**DOI:** 10.1590/2317-1782/20232021197pt

**Authors:** Josilene Luciene Duarte, Kelly da Silva, Fabiana Cristina Carlino, Maria Victória dos Anjos Souza, Greicielly da Silva Pereira Vieira, Ana Maria Carregosa, Sulamita Cysneiros das Chagas Santos

**Affiliations:** 1 Departamento de Fonoaudiologia, Universidade Federal de Sergipe - UFS - Lagarto (SE), Brasil.; 2 Clínica Otocenter - Aracaju (SE), Brasil.

**Keywords:** Triagem Neonatal, Perda Auditiva, Diagnóstico Precoce, Reabilitação, Indicadores de Qualidade em Assistência à Saúde

## Abstract

**Objetivo:**

Descrever o panorama da saúde auditiva infantil no Sistema Único de Saúde do estado de Sergipe.

**Método:**

Estudo quantitativo e retrospectivo, composto por quatro etapas: 1) Busca no Cadastro Nacional de Estabelecimento de Saúde das instituições conveniadas ao Sistema Único de Saúde no estado de Sergipe que realizam serviços obstétricos e dos serviços que atuam na saúde auditiva; 2) Obtenção de dados de cobertura da Triagem Auditiva Neonatal (TAN), por meio do DATASUS (de 2012 a 2020); 3) Coleta de dados em prontuários das instituições com obstetrícia e/ou que realizam a TAN; e 4) Entrevista aos responsáveis das crianças em reabilitação auditiva. Os resultados foram sumarizados por meio de estatística descritiva (frequência absoluta e relativa, medidas de tendência central e de dispersão).

**Resultados:**

Dos 29 estabelecimentos com obstetrícia, um realiza a TAN. Há dois Centros de Referência em Saúde Auditiva (CRSA) com habilitação para implante coclear e dois Centros Especializados em Reabilitação. De 2012 a 2020 a cobertura da TAN no estado foi inferior a 40% e quando realizada na maternidade, houve ausência de encaminhamentos para a realização do Potencial Evocado Auditivo de Tronco Encefálico (PEATE) e do diagnóstico audiológico. Observou-se cobertura considerável no CRSA com menor taxa de evasão para realizar PEATE e com taxa de diagnóstico de 4,8%. O tempo médio da TAN universal à reabilitação foi superior ao recomendado.

**Conclusão:**

Existe necessidade de aumentar a cobertura da TAN, ajustar a rede de saúde auditiva para a articulação nos diferentes níveis de atenção e diminuir o tempo para identificação, diagnóstico e início da reabilitação.

## INTRODUÇÃO

As políticas públicas em saúde auditiva vêm sofrendo avanços nos últimos anos com o objetivo de favorecer o diagnóstico e a reabilitação da deficiência auditiva precocemente. Com o intuito de padronizar os Indicadores de Risco para a Deficiência Auditiva (IRDA) e alcançar os objetivos dos programas de saúde auditiva, que inclui o desenvolvimento da linguagem oral de forma plena, o Comitê Multiprofissional em Saúde Auditiva (COMUSA)^(1)^, baseado nas normativas internacionais do *Joint Committee on Infant Hearing* (*JCIH*)^(2)^ do ano de 2007, criou em 2010 os indicadores de qualidade para a Triagem Auditiva Neonatal (TAN).

Estes Indicadores, serviram como base para a criação em 2012, pelo Ministério da Saúde, das Diretrizes de Atenção da Triagem Auditiva Neonatal^(3)^. Em 2020, após a atualização do documento do *JCIH* ^(4)^ (2019), o COMUSA^(5)^ publicou uma nota técnica inserindo novos IRDA e enfatizando as recomendações já existentes da realização dos procedimentos de Emissões Otoacústicas Evocadas (EOAE) em crianças sem IRDA e do Potencial Evocado Auditivo de Tronco Encefálico - Automático (PEATE-A), em crianças com indicadores de risco.

Esta atualização inseriu metas mínimas de cobertura da TAN, com realização em 95% dos neonatos antes da alta hospitalar ou no máximo até o primeiro mês de vida; reteste da TAN, quando necessário, após 15 dias da alta hospitalar; índice menor que 4% de encaminhados para diagnóstico; conclusão do diagnóstico até o terceiro mês de vida em 90% dos casos e início de amplificação sonora, em 95% dos casos de deficiência auditiva bilateral permanente, em até um mês após a confirmação do diagnóstico e início da reabilitação auditiva entre o terceiro e sexto mês de vida da criança^(1-6)^.

Estudos nacionais realizados em diferentes regiões do país evidenciam a necessidade de aumento da cobertura da TAN e cumprimento das metas estabelecidas, além de apontar para uma disparidade regional, com melhores resultados no sudeste e sul do país e piores no Norte e Nordeste^(7-12)^.

Ainda, estudos nacionais verificaram que no período de 2008 a 2011 houve um crescimento de 208% na cobertura da TAN e as regiões que obtiveram maior crescimento foram as regiões Sul e Nordeste^(13)^, sendo a região Sul com índice superior ao estimado (189%) e com maior aumento proporcional (65%) e a região Norte com metade da cobertura (54,5%) e mesmo assim despontando como o segundo maior aumento (58%)^(14)^, o que indica que antes deste aumento a cobertura era muito abaixo do restante do país. As regiões Norte e Nordeste, até 2015, apresentavam maior percentual de locais sem qualquer cobertura da TAN^(6)^.

Neste sentido fica evidente a necessidade de estudos que demonstrem a organização da rede de saúde auditiva para a população pediátrica em diferentes regiões do Brasil, evidenciando se as normativas estão sendo respeitadas e, discutindo possibilidades de melhoria e entraves encontrados. Portanto, o objetivo deste estudo é descrever o panorama da saúde auditiva infantil no Sistema Único de Saúde (SUS) do estado de Sergipe.

## MÉTODO

Trata-se de um estudo com abordagem quantitativa, retrospectivo, transversal, de natureza analítica, com a aprovação do Comitê de Ética em Pesquisa (Parecer de número 4.293.898; CAAE 92530218.7.0000.5546). A pesquisa foi composta por quatro etapas: 1) Busca no CNES das instituições conveniadas ao SUS no estado de Sergipe que realizam serviços obstétricos e busca das instituições que atuam na saúde auditiva, em todos os níveis de complexidade; 2) Obtenção de dados de cobertura da TAN, por meio do DATASUS, no período de 2012 a 2020; 3) coleta de dados em prontuários das instituições com obstetrícia e/ou que realizam a TAN; e 4) Entrevista aos pais e/ou responsáveis das crianças que estão em reabilitação auditiva.

### Busca no CNES das instituições conveniadas ao SUS

Inicialmente foi realizada uma busca ativa no Cadastro Nacional de Estabelecimentos de Saúde (CNESNet), por meio do endereço do CNES^(15)^, para conhecimentos dos estabelecimentos de saúde conveniados ao SUS que realizam atendimentos obstétricos (que consequentemente deveriam ofertar a Triagem Auditiva Neonatal Universal -TANU) e os estabelecimentos de saúde que realizam ações voltadas à saúde auditiva desde a atenção básica a atenção especializada.

### Obtenção de dados de cobertura da TAN por meio do DATASUS

Foi realizada uma busca no DATASUS, considerando o período de 2012 (ano de início da TAN, no estado de Sergipe) a 2020. O quantitativo de Neonatos Nascidos Vivos (NNV), foi possível por meio do Sistema de Informações sobre Nascidos Vivos - SINASC e o quantitativo de TAN realizadas foi possível por meio do Sistema de Informações Ambulatoriais (SIA-SUS). Para a realização das buscas no SIA-SUS foram utilizados os códigos de procedimento apresentados na Tabela 1.

**Tabela 1:** Códigos de procedimentos utilizados na busca no Sistema de Informações Ambulatoriais do Sistema Único de Saúde (SIA-SUS)

Procedimentos pesquisados	Código dos procedimentos
Emissões otoacústicas evocadas para a triagem auditiva (teste da orelhinha)	0211070149
Potencial Evocado Auditivo para a triagem auditiva (teste da orelhinha)	0211070270
Avaliação para o diagnóstico diferencial da deficiência auditiva*	0211070106
AASI externo retroauricular tipo C^1^^*^	0701030143
Cirurgia de Implante Coclear Unilateral*	0404010571
Cirurgia de Implante Coclear Bilateral	0404010580

* Indica procedimentos realizados também em grupos específicos, com mais de três anos de idade. ^1^Indicado para a faixa etária infantil devido a versatilidade proporcionado pelos algoritmos e características eletroacústicas.

A cobertura da TAN no estado foi calculada por meio dos dados coletados no DATASUS, considerando o quantitativo de Neonatos Nascidos Vivos (NNV) subtraído do quantitativo de neonatos que realizaram a TAN (por meio de Emissões Otoacústicas ou Potencial Evocado Auditivo de Tronco Encefálico (PEATE) (NNV- (TAN EOA + TAN PEATE). Já o percentual da cobertura da TAN foi calculado pela fórmula ([TAN EOA + TAN PEATE] / NNV) * 100.

### Obtenção de dados de prontuários

Após a coleta dos estabelecimentos de saúde pelo CNESnet, a Secretaria Estadual de Saúde, indicou quatro instituições credenciadas ao SUS que efetivamente realizam os cuidados à Saúde Auditiva no estado de Sergipe: a Associação de Assistência e Proteção Materno-Infantil de Sergipe (Maternidade Zacarias Junior), o Centro Especializado em Reabilitação de Sergipe (CER III Dona Maroca), o Centro de Referência em Saúde Auditiva - CRSA (Hospital São José) e o Hospital Universitário da Universidade Federal de Sergipe no Campus de São Cristóvão (HU-UFS).

Foi encaminhada uma carta convite aos gestores destas quatro instituições, além do TCLE e um formulário desenvolvido no google formulários para a coleta das informações referentes aos quantitativos de TAN, diagnóstico auditivo, concessão de dispositivos eletrônicos auditivos e terapia fonoaudiológica. Três serviços aceitaram participar da pesquisa, a Associação de Assistência e Proteção Materno-Infantil de Lagarto (Maternidade Zacarias Junior), o CER III e o CRSA (Hospital São José). O CER III foi excluído da pesquisa por ter sido habilitado recentemente e não possuir um fluxo de atendimento na área da deficiência auditiva. Desta forma, foram analisados 3.741 prontuários na maternidade e 11.400 no CRSA.

O percentual de evasão a TAN na maternidade foi calculado considerando a fórmula (Neonatos que não realizaram a TAN / Número total de prontuários analisados)*100. Já no CRSA, o percentual foi calculado por (neonatos que não retornaram para reteste da TAN / Número total de neonatos que realizaram a TAN) * 100.

### Obtenção de dados primários por meio de entrevista telefônica

Os dados referentes ao tempo médio decorrente entre o processo da TAN à reabilitação foram obtidos a partir dos dados de 25 indivíduos que se encontravam em processo de terapia fonoaudiológica no CRSA. Foi realizada uma entrevista por telefone, em que os pais foram orientados sobre os objetivos do estudo e concederam as informações sobre livre e espontânea vontade, após a leitura pelo entrevistador acerca da carta de informação ao sujeito da pesquisa e o TCLE.

Os pais foram questionados a respeito da idade da realização da TAN, o tempo entre o teste e reteste da TAN, a idade em que compareceu ao primeiro atendimento para iniciar o diagnóstico audiológico (no centro de referência), o tempo entre o início e o fim do diagnóstico, a idade em que compareceu ao primeiro atendimento para o processo de seleção do dispositivo eletrônico auditivo, o tempo entre o diagnóstico e a seleção do dispositivo, a idade em que foi adaptado o dispositivo, o tempo entre a seleção e a adaptação do dispositivo e a idade em que iniciou o processo de terapia fonoaudiológica.

### Tabulação e análise dos dados

Os dados foram inseridos em planilhas do Microsoft Office Excel. Para sumarização dos resultados foi realizada a estatística descritiva por meio do cálculo das frequências relativas e absolutas para os resultados categóricos; medidas de tendência central (média) e de dispersão (desvio padrão) para os dados quantitativos; e estimativa de indicador de cobertura e evasão da TAN.

## RESULTADOS

### Busca no CNES das instituições conveniadas ao SUS

Os dados coletados no CNESNet para o estado de Sergipe mostraram 29 estabelecimentos que ofertam os serviços obstétricos pelo SUS. Destes, somente dois (6,9%) realizam a TAN (uma no município de Lagarto e outra em Aracaju). Os neonatos nascidos em duas maternidades públicas da capital (Nossa Senhora de Lourdes e Santa Izabel) são encaminhados para realizar a TAN nos dois únicos CRSA habilitados para a realização do implante coclear (um no Hospital São José e outro no Hospital Universitário da Universidade Federal de Sergipe). Ainda há no estado dois CER, o CER IV na regional de Aracaju e o CER III na regional de Lagarto, para realizar desde a triagem à reabilitação auditiva, com a concessão de Aparelho de Amplificação Sonora Individual (AASI).

### Obtenção de dados de cobertura da TAN por meio do DATASUS

A Tabela 2 apresenta os dados coletados no DATASUS referentes aos quantitativos dos NNV, das triagens auditivas neonatais, da avaliação auditiva para a faixa etária de estudo, da concessão de dispositivos eletrônicos auditivos para a faixa etária do presente estudo, para o estado de Sergipe, considerando o período de 2012 a 2020. Chamando a atenção para o fato de que para a avaliação auditiva e para a concessão de dispositivos eletrônicos auditivos, não é possível distinguir entre faixas etárias, já que os códigos em que incluem o grupo inferior a três anos, se aplica a procedimentos que podem ser realizados em faixa etária superior a esta. Neste caso, a porcentagem foi apresentada somente para a TAN.

**Tabela 2:** Panorama da atenção à saúde auditiva infantil no estado de Sergipe, com base no DATASUS

Ano	NNV	TAN EOAE (sem risco) (n, %)	TAN PEATE (com risco) (n, %)	Total TAN (% realizada)	Avaliação p/ Diagnóstico^*^ (n)	AASI tipo C^**^ (n)	IC^***^ (n)		
2012	27.354	3.801 (13,9%)	200 (0,7%)	4.001 (14,6%)	25	174	----	----	----
2013	27.183	5.999 (22,1%)	67 (0,2%)	6.066 (22,3%)	26	258	----	----	----
2014	27.502	8.523 (31%)	224 (0,8%)	8.747 (31,8%)	48	169	13	----	13
2015	27.797	8.693 (31,3%)	183 (0,7%)	8.876 (31,9%)	172	88	18	----	18
2016	25.702	8.240 (32,1%)	118 (0,5%)	8.358 (32,5%)	56	88	17	----	17
2017	33.867	8.764 (25,9%)	121 (0,4%)	8.885 (26,2%)	131	101	16	7	23
2018	34.256	11.536 (33,7%)	115 (0,3%)	11.651 (34,0%)	27	54	7	6	13
2019	32.697	8.599 (26,3%)	146 (0,4%)	8.745 (26,7%)	51	88	7	7	14
2020	31.784	6,393 (20,1%)	208 (0,7%)	6.601 (20,8%)	18	87	6	2	4
2012-2020	240.788	70.548 (29,3%)	1.382 (0,6%)	71.930 (29,9%)	554	1107	68#	22##	102###

* Código SUS 0211070106 (avaliação para diagnóstico diferencial da deficiência auditiva), que inclui a avaliação para crianças menores de 3 anos;

** AASI tipo C é referente ao tipo de tecnologia a qual é a indicada para crianças;

*** IC com indicação pediátrica e adulto;

# código SUS 0404010148 (implante coclear);

## código SUS 0404010571 (cirurgia de implante coclear unilateral);

### código SUS 0404010580 (cirurgia de implante coclear bilateral)

Legenda: NNV = neonatos nascidos vivos; TAN = triagem auditiva neonatal; EOAE = emissões otoacústicas evocadas; PEATE = potencial evocado auditivo de tronco encefálico; n = número de casos; % = porcentagem de casos; AASI = aparelho de amplificação sonora individual; IC = implante coclear

Baseado nos dados do DATASUS de 2012 a 2020, a cobertura de TAN nos NNV foi de 27,7%.

### Obtenção de dados de prontuários

Na maternidade os dados foram coletados de 2013 a 2020, com média mensal de 210 NNV. Já no CRSA foram coletados dados referentes aos anos de 2018 a 2020. A Figura 1 apresenta um fluxograma dos prontuários analisados na maternidade de estudo, de acordo com os desfechos da TAN.

**Figura 1:** Valores absolutos e porcentagens de prontuários analisados na maternidade da regional de Lagarto (SE), de acordo com o fluxo de eventos do Programa de TAN realizada somente EOAE-T, no período de janeiro/2013 a outubro/2020

Legenda: TAN = Triagem Auditiva Neonatal; EOET = Otoemissão por estímulo transiente; IRDA = Indicador de Risco para a Deficiência Auditiva; PEATE = Potencial Evocado Auditivo de Tronco Encefálico

Os dados coletados demonstram que a cobertura da TAN na maternidade, considerando o período de 2013 a 2020, foi de 67,4%. Uma análise dos dados dos três últimos meses demonstrou que, do total de 625 NNV, 36 (5,8%) não realizaram a TAN, o que demonstra uma cobertura de 94,2%. Todos os neonatos realizaram a TAN com EOAE por transiente (EOAE-T) e não constam encaminhamentos para a realização do PEATE nos que apresentaram IRDA (6,4% dos NNV no período de estudo).

A Figura 2 apresenta um fluxograma dos prontuários analisados no CRSA, de acordo com os desfechos da TAN.

**Figura 2:** Valores absolutos e porcentagens de neonatos triados com EOAE-T e PEATE no Centro de Referência em Saúde Auditiva (CRSA) na regional de Aracaju (SE), para o período de abril/ 2018 a março/2020

Legenda: TAN = Triagem Auditiva Neonatal; EOET = Otoemissão por estímulo transiente; IRDA = Indicador de Risco para a Deficiência Auditiva; PEATE = Potencial Evocado Auditivo de Tronco Encefálico

Assim como na maternidade, a TAN é realizada no CRSA por meio de emissões otoacústicas evocadas por estímulos transientes EOAE-T. Para a realização do PEATE em neonatos com IRDA é agendado retorno para a realização do PEATE, independente do resultado da TAN. As crianças que falham na TAN, mas que não têm IRDA, retornam para o reteste apenas com EOAE-T. A taxa de evasão no retorno para realizar o PEATE em neonatos com IRDA foi de 45,8% e a evasão dos que falharam na TAN e não apresentaram IRDA foi de 0,9%.

A taxa total de evasão para a TAN na maternidade após a alta hospitalar foi, de 2012 a 2020, de 32,6%. Quando a TAN foi realizada no CRSA, a taxa total de evasão para o retorno da TAN foi de 0,6%.

### Obtenção de dados primários por meio de entrevista

Foi realizada uma entrevista por telefone para os pais/responsáveis de 25 pacientes que estavam em processo de terapia fonoaudiológica para a reabilitação auditiva no CRSA no ano de 2020. Neste tipo de análise, não foi possível respeitar o período inicial de 2012, pois muitas crianças que estavam em terapia neste serviço, nasceram em datas inferiores a esta. A Tabela 3 apresenta o resultado de 25 pacientes

**Tabela 3:** Idade dos pacientes em uso de dispositivos (Implante Coclear e/ou Aparelho de Amplificação Sonora Individual (AASI) no momento de realização da TAN, do diagnóstico, da adaptação de dispositivo eletrônico e do início da terapia fonoaudiológica no Centro de Referência em Saúde Auditiva

		IDADE no momento da(o)				DISPOSITIVO
ID	DN	TAN	Diagnóstico	Adaptação	Terapia	
1	04/11/04	NR	168	180	180	IC
2	06/05/06	NR	30	84	72	AASI e IC
3	27/07/07	22	22	23	23	AASI e IC
4	13/10/07	4	10	24	36	AASI e IC
5	14/11/08	16	16	18	16	AASI e IC
6	02/02/09	16	18	20	112	IC
7	07/07/11	Nascimento	19	32	24	AASI e IC
8	22/10/11	NR	24	36	36	IC
9	07/11/11	Nascimento	15	15	17	AASI e IC
10	28/12/11	NR	17	20	20	IC
11	16/01/12	NR	48	48	48	AASI e IC
12	30/09/12	NR	20	36	36	AASI e IC
13	26/11/13	Nascimento	19	48	36	AASI e IC
14	08/01/14	Nascimento	20	36	36	AASI
15	01/03/15	Nascimento	1	2	4	IC
16	18/04/15	18	24	27	38	AASI e IC
17	14/12/15	12	12	18	18	IC
18	21/01/16	Nascimento	12	18	37	IC
19	09/04/16	12	16	36	24	IC
20	26/08/16	NR	36	36	3	AASI
21	22/10/16	22	22	29	24	AASI
22	22/02/17	4	24	24	24	IC
23	14/04/18	2	12	19	19	AASI
24	18/04/18	8	8	29	29	AASI
25	24/05/19	4	24	28	28	AASI

Legenda: ID = identificação; DN = data de nascimento; NR = não realizou; TAN = triagem auditiva neonatal; IC = Implante Coclear; AASI = Aparelho de Amplificação Sonora Individual

Do total de 25 indivíduos, 15 nasceram a partir de 2012, período em que a TAN teve início no estado de Sergipe, sendo que destes, três não realizaram a TAN e foram encaminhados da atenção básica para o CRSA para avaliação audiológica por apresentarem queixa familiar de atraso no desenvolvimento da função auditiva e de linguagem. Observando o panorama de cada etapa do programa de saúde auditiva para este grupo de indivíduos, considerando o período de 2012 a 2020, o tempo médio (±desvio padrão) em meses, para a realização de cada etapa foi de 6,8 (±7,6) para a TAN; 16,1 (±7,3) para o diagnóstico; 26,1 (±11,5) para a concessão dos dispositivos eletrônicos auditivos e 26,4 (±9,9) para o início da terapia fonoaudiológica.

## DISCUSSÃO

Os principais resultados desta pesquisa indicam no Estado Sergipe uma cobertura da TAN e taxas de evasão acima do recomendado, com tempo elevado entre o diagnóstico e a reabilitação auditiva, indicando desafios a serem enfrentados pelos serviços públicos.

A Atenção à Saúde Auditiva no estado de Sergipe, da TAN à reabilitação, teve início no ano de 2012, sendo a TAN inicialmente realizada apenas pelo CRSA. O estado é dividido em sete regionais de saúde, sendo a TAN ofertada quase que em sua totalidade pelas regionais de Aracaju e Estância, Propriá e Nossa Senhora do Socorro (NSS), também aderiram a realização da TAN, porém os registros no DATASUS demonstram baixa cobertura no ano de 2020. Estes dados demonstram que há desigualdade na oferta deste serviço no Estado.

Dos 29 estabelecimentos que ofertam a obstetrícia, somente uma maternidade oferta a TAN (na cidade de Lagarto) e os dois únicos CRSA do estado, que estão localizados na regional de Aracaju, recebem neonatos apenas de duas maternidades da Regional de Aracaju. Neste sentido, os neonatos nascidos nas demais maternidades, não são encaminhados para a realização da TAN. Dados do DATASUS alertam que embora nasçam aproximadamente 2.500 crianças por mês no estado, apenas são realizadas 700 TAN por mês, evidenciando uma baixa cobertura da TAN nesta região do Brasil^(6)^. Contrariamente ao que se esperava^(13)^, não foi observado o aumento desta cobertura ao longo dos anos no estado de Sergipe.

Ainda com base nos resultados encontrados no DATASUS, a cobertura da TAN está muito abaixo do preconizado e representa o maior desafio deste estado em cumprir as normativas^(1-5)^ das políticas públicas em saúde auditiva. Isto porque a TAN é a porta de entrada para a efetividade do programa.

Na análise geral dos resultados ficou evidente que para alcançar os objetivos das políticas públicas em saúde auditiva propostas pelo ministério da saúde, existe a urgência de credenciamento de mais unidades e/ou maternidades para a realização da TAN no estado, pois a cobertura que variou de 14,0% à 34,0% de 2012-2020, ainda é muito inferior ao recomendado, que é de 95,0% dos NNV^(1,5)^. Além disso, é importante ressaltar a importância destes centros estarem articulados com os CRSA e com os CERs, para que ocorra o encaminhamento e agendamento dos neonatos que necessitam de acompanhamento e diagnóstico audiológico.

Os equipamentos indicados para a realização da TAN são Emissões Otoacústicas Evocadas e Potencial Evocado Auditivo de Tronco Encefálico^(2-5)^. Desde 2012 o PEATE foi elencado pelo Ministério da Saúde como obrigatório em neonatos que apresentam IRDA^(3)^. Neste sentido, muitas instituições que realizam a TAN ainda possuem somente as EOAE, seja por estímulos transientes (EOAE-T) ou por produto de distorção (EOAE-PD), já que o custo do equipamento de PEATE é elevado, o que dificulta a sua aquisição por algumas instituições públicas^(12)^. Uma estratégia para este problema seria o encaminhamento dos neonatos com IRDA para instituições credenciadas ao SUS, que realizam o PEATE e que fazem parte da rede especializada.

Em relação à realização da TAN, tanto na maternidade como no CRSA, é utilizada as EOAE-T, sendo que no CRSA, os neonatos com IRDA, independente do resultado da TAN (Passa ou Falha), comparecem em um retorno na instituição para a realização do PEATE. Já os neonatos sem IRDA, mas com falha na TAN, realizam o reteste com EOAE-T. Estes achados corroboram com alguns estudos da literatura em que a TAN é realizada com EOAE-T, independente da condição do neonato^(12,16)^. Entretanto, é importante destacar que as EOAE-T não são sensíveis para detectar perdas auditivas retrococleares, assim este tipo de alteração auditiva pode não estar sendo detectada de forma precoce, o que contraria os objetivos da TAN^(1-5)^.

Este fato é reforçado pela falta de encaminhamento para a realização do PEATE para os neonatos com IRDA que nasceram na maternidade. Além disso, no CRSA, dos neonatos com IRDA que passaram na TAN com EOAE-T e retornaram para realizar o PEATE, a maioria falharam, reforçando a hipótese da possibilidade de neonatos com alteração retrococlear neste grupo. Esta hipótese poderia ser confirmada se houvesse o acesso à avaliação diagnóstica, o que não foi possível, dada a amostra numerosa e a falta do banco de dados digitalizado pela instituição.

Também é importante chamar a atenção para a realização do PEATE já no primeiro teste de TAN quando o neonato apresenta IRDA, pois a taxa de evasão para a realização do PEATE nestes neonatos com resultado passa na EOAE-T é de quase metade dos casos, uma evasão significantemente maior do que quando a criança falha nas otoemissões.

Outro achado que chamou a atenção quando a TAN foi realizada na maternidade, foi o alto índice do resultado “passa” com EOAE-T associado ao não encaminhamento para realizar o PEATE em caso de IRDA, para o diagnóstico e para o acompanhamento audiológico. A literatura descreve que mesmo os programas que conseguem realizar a TAN de forma universal, apresentem taxa de encaminhamento para diagnóstico em torno de 0,3 a 1,8%^(7,11,16)^, sendo recomendado não ultrapassar 4,0%^(1,3)^.

Cabe ressaltar que programas que atingiram taxa de adesão a TAN na maternidade similares ao deste estudo (em torno de 65,0%) apresentaram taxa de encaminhamento em torno de 2,0%^(8,9)^. Assim, embora a maternidade de Lagarto cumpra a lei nº 12.303^(17)^ não há a operacionalização necessária para estar inserida em um contexto de Saúde Auditiva.

Diferentemente, no CRSA a taxa de resultado “passa” foi de aproximadamente 86,0%, corroborando com outros estudos que apresentam dados em torno de 75,6 a 88,3%^(18-20)^ e de encaminhamento para acompanhamento por IRDA de 0,3% e diagnóstico 4,8%

A taxa de evasão observada neste estudo foi similar aos encontrados na literatura, que variou de 9,0% à 40,0% para diferentes estudos^(7,10,11,21)^. Já quando a TAN foi realizada no CRSA, a taxa de evasão para o retorno foi de 0,7%, possivelmente devido a compreensão dos pais/responsáveis da necessidade da realização do exame para a detecção precoce da deficiência auditiva é um fator a ser considerado dentro do programa^(22)^, ficando evidente que a orientação e o contato com a família do neonato independentemente do local de realização da TAN, pode ajudar a diminuir as taxas de evasão a TAN e ao programa como um todo.

Todos os centros que foram obtidas a anuência para a realização da pesquisa, não dispunham de banco de dados. Neste sentido, não foi possível realizar o gerenciamento do programa de saúde auditiva. Este achado corrobora com os achados da literatura para programas de diferentes regiões do país^(12)^. De acordo com o COMUSA^(1)^ e Ministério da Saúde^(3)^, o banco de dados favorece as medidas dos indicadores de qualidade e demostra o panorama do programa de saúde auditiva no que diz respeito às porcentagens de reteste, encaminhamentos para acompanhamento da função auditiva, diagnóstico audiológico, concessão dos dispositivos eletrônicos auditivos e terapia fonoaudiológica.

Ainda devido a falta do banco de dados, para verificar se os programas de saúde auditiva estavam atendendo aos critérios internacionais para minimizar os efeitos da privação sensorial na neuroplasticidade do sistema auditivo, foi realizada entrevista para a aplicação de um questionário com pais/responsáveis, via telefone com o intuito de verificar o tempo entre as etapas desde a TAN a terapia fonoaudiológica. A proposta inicial seria coletar informações desde 2012 (ano de início da TAN no estado), entretanto, na instituição de coleta só havia 25 indivíduos inscritos realizando a terapia fonoaudiológica, sendo necessário avaliar todos os períodos. De acordo com o Ministério da Saúde^(3)^ e COMUSA^(1,5)^ a TAN (teste e reteste) deve ser realizada no primeiro mês de vida, o diagnóstico até o terceiro mês e a reabilitação deve ter início até o sexto mês. Nossos dados demonstraram que o tempo entre as etapas, é muito superior ao recomendado.

Sabe-se que a avaliação para o diagnóstico da deficiência auditiva, demanda de várias etapas de procedimentos e que muitas vezes não é passível de conclusão em uma única sessão, havendo uma demora nesta etapa do processo. O mesmo pode ocorrer da etapa do teste para a seleção e concessão dos dispositivos eletrônicos auditivos. Entretanto, de acordo com os dados deste estudo, fica evidente que, uma vez que o neonato chega a este nível de cuidado, não existe uma demora considerável para o diagnóstico, com maior tempo despendido na etapa de teste/seleção/adaptação dos dispositivos eletrônicos auditivos.

Este tempo elevado é justificável, pois muitas vezes o SUS não tem os dispositivos disponíveis prontamente. Para mitigar os danos causados pela privação sensorial neste período de aquisição das habilidades auditivas e de linguagem, este serviço insere o paciente em terapia, antes mesmo da concessão dos dispositivos eletrônicos auditivos.

É importante ressaltar que para o sucesso das políticas públicas em saúde auditiva é necessário um programa de saúde auditiva bem estruturado, para não comprometer o objetivo principal da TAN e do programa de saúde auditiva que é a detecção, diagnóstico e a intervenção precoce, com a finalidade do desenvolvimento da função auditiva e de linguagem dentro do período de maior plasticidade neuronal, para o desenvolvimento destas funções como ocorre na criança normo-ouvinte.

É importante reforçar que o investimento em políticas públicas em saúde auditiva prioriza a oralidade, sendo que a língua de sinais só será uma alternativa para as famílias que não tem interesse no desenvolvimento da oralidade, seja por fatores familiares e/ou culturais, ou para aqueles indivíduos com comorbidades que inviabilizam o desenvolvimento da linguagem oral.

Quanto às limitações do estudo, a falta de banco de dados para gerenciamento e operacionalização do programa de saúde auditiva local, associado ao número escasso de informações nos prontuários, dificultou a coleta das informações necessárias para medir os indicadores de qualidade dos serviços para este estado, de forma longitudinal. São necessários estudos longitudinais que acompanhem os neonatos de maneira individualizada, da TAN à reabilitação auditiva, com o intuito de verificar as reais dificuldades dos programas nesta região e fomentar ações e soluções que levem o estado evoluir nas políticas públicas de saúde.

## CONCLUSÃO

O panorama da saúde auditiva infantil no estado de Sergipe, descreve ações nos diferentes níveis de atenção, porém com necessidade de ajustes para a detecção e tratamento da deficiência auditiva de forma precoce. A cobertura da TAN encontra-se significativamente abaixo do preconizado pelos órgãos competentes, necessitando da oferta nos diferentes municípios do estado e aumento no investimento em tecnologias, já que a maioria das TANs são realizadas somente com EOAE. Além disso, a rede de atenção em saúde auditiva deve ser articulada, favorecendo o acesso do paciente nos diferentes níveis de atenção, diminuindo o tempo para identificação, diagnóstico e início da reabilitação, que neste estudo foi superior ao recomendado.
